# 23 Gauge pars plana vitrectomy for the removal of retained intraocular foreign bodies

**DOI:** 10.1186/s12886-015-0067-2

**Published:** 2015-07-16

**Authors:** Kemal Yuksel, Ugur Celik, Cengiz Alagoz, Huseyin Dundar, Burcu Celik, Ahmet Taylan Yazıcı

**Affiliations:** Department of Ophthalmology, Beyoglu Eye Training and Research Hospital, Istanbul, Turkey; Department of Ophthalmology, Gaziosmanpaşa Taksim Training and Research Hospital, Istanbul, Turkey; Department of Ophthalmology, Diyarbakir Ergani Government Hospital, Diyarbakir, Turkey; Merkezefendi Mah. Mevlana Cad. Sedeftepe Evleri. Blok:96 No:26, Zeytinburnu, Istanbul Turkey

**Keywords:** Pars plana vitrectomy, Intraocular foreign body, Posterior segment surgery, Penetrating ocular trauma

## Abstract

**Background:**

To evaluate the morpho-functional outcomes and safety of transconjuctival 23-gauge pars plana vitrectomy(PPV) for removal of intraocular foreign bodies (IOFBs).

**Methods:**

A retrospective study of 36 consecutive cases (mean age; 34,2 ± 10,9 years (between 15 and 60), 27 M,9 F) of 23-G PPV for the removal of IOFBs during the period of April 2009 and December 2011 and followed 9,4 ± 6,4(2–27) months were conducted. Visual outcomes, slit lamp biomicroscopy, intraocular pressure (IOP), and posterior segment visualization by indirect ophthalmoscopy, A-B mode ultrasonography, and computed orbital tomography were performed for all cases. Main outcomes including anatomic and visual outcomes, and both intraoperative and postoperative complications were recorded.

**Results:**

Of the 36 cases available for the study, the IOFBs (size range, 3 to 12 mm) could be removed in all eyes. Mean preoperative LogMAR BCVA was 1.44 ± 138 (range, 1.00 to 0.00) and mean postoperative LogMAR BCVA at final visit was 0,78 ± 0,98 (range, 1.00 to 0.00). (*P* = 0,007) Anatomic success was obtained in 97.2 % of eyes. 16 patients needed primary wound repair due to the leakage in insertion sites before the PPV, however remaining 20 cases were not. Fibrin reaction was seen in 8 (22.2 %) patients in early postoperative period, intraocular pressure elevation was detected in 12 (33.3 %) patients in which the silicone oil was used as an intravitreal tamponade, one patient with silicone oil tamponade developed band keratopathy and phthisis bulbi.

**Conclusions:**

23-Gauge PPV is a feasible, effective approach in the surgical management of the patients with posterior segment intraocular foreign bodies.

## Background

Penetrating ocular injury with an associated retained intraocular foreign body (IOFB) is an important cause of blindness and ocular morbidity. It is encountered in 17–41 % of open globe injuries [1–4]. Ocular injuries caused by IOFBs are often associated with corneal and scleral penetrating injury, hypheama, vitreous hemorrhage, lens injury, retinal damage or detachment, and even more serious complications such as endophthalmitis [5–7]. Besides the initial damage, ocular abnormalities caused by surgical intervention and postoperative complications can lead to poor visual outcomes.

There are many methods or techniques to remove IOFBs [8, 9]. The surgical approaches for posterior segment foreign bodies include vitrectomy and removal with the help of magnet or forceps. The aim of the treatment is to restore the ocular integrity and obtain good visual outcomes. Recent advances in vitreoretinal surgery and microsurgical techniques have improved the success rate of pars plana vitrectomy (PPV) in the management of ocular injuries with retained posterior segment IOFB [10–13].

Pars plana vitrectomy results of the removal of posterior segment IOFBs, have been reported before [14–16]. Removal of posterior segment IOFBs by vitrectomy is advocated because it provides direct viewing and controlled removal of the IOFB [17]. Vitrectomy, by the removal of blood in the vitreous, prevents inflammatory and fibrous responses that may lead to tractional sequel in the posterior segment [18, 19] and allows an improved view of the retina facilitating treatment of retinal breaks. A possible reduced risk of endophthalmitis has been suggested when pars plana vitrectomy is performed for the removal of IOFBs [20]. Even numerous publications related with intraocular foreign bodies the 23 gauge PPV and incision for IOFB removal has not studied in the literature [21–23]. The aim of the current study was to evaluate the anatomic and visual outcomes and safety of transconjuctival 23-gauge PPV for removal of IOFBs.

## Methods

### Study design and subjects

In this retrospective study, 36 eyes of 36 patients who underwent 23-G transconjuctival PPV for the removal of IOFB in Beyoglu Eye Education and Research Hospital, in Istanbul, Turkey between April 2009 and December 2011 were included to the study group. Informed consent was obtained from all of the subjects before the surgery. The informed consents were written. The principles of the study were obeyed to the declaration of Helsinki and were approved by the Beyoglu Eye Education and Research Hospital’s local ethic committee. Advancement in microsurgical techniques and successful surgical results of 23 Gauge pars plana vitrectomy in posterior segment IOFB patients were the main rationalities of this study.

### Examination protocol

Preoperative, intraoperative, and postoperative examinations were retrospectively evaluated from medical records, including: demographic information, examination details including Snellen best corrected visual acuity (BCVA), slit lamp biomicroscopy, intraocular pressure (IOP) measurement using applanation tonometry, and posterior segment visualization by indirect ophthalmoscopy. Determination of the localization and size of the IOFB and its association with orbita, orbital radiographs, A-B mode ultrasonography, and computed orbital tomography were carried out.

### Surgical procedures

#### 23 G PPV and IOFB removal and phacoemulsification

Surgeries were performed under general anesthesia, and phacoemulsification was performed prior to PPV. Phacoemulsification was performed via a 2.8 mm clear corneal tunnel with a standard phaco-chop technique. A foldable hydrophilic acrylic IOL was implanted in the bag in the event that the posterior capsule was intact. In cases with posterior capsule defects, a polymethylmethacrylate IOL with an overall diameter of 13 mm was placed in the ciliary sulcus. The incision was sutured with 10-0 nylon.

#### 23 G PPV and IOFB removal

Surgery was performed under general anesthesia or local anesthesia. The conjunctiva was displaced using a special pressure plate (DORC, Zuidland, Holland) over the intended sclerotomy sites. The cannula was then inserted at a 10–30° angle 3.5 mm from the limbus through the conjunctiva, sclera, and pars plana. The cannulas were placed in the inferotemporal, superotemporal, and superonasal quadrants. The cannulas were inserted using beveled trocars, a single-step procedure. An illumination probe was placed at the superonasal quadrant, and a 23-G infusion cannula was placed at the inferotemporal quadrant. A noncontact Biom indirect viewing system (Oculus Inc., Petaluma, CA) was used for visualization of the posterior segment.

All surgical procedures were carried out using the 3-port, 23-gauge vitrectomy system from Alcon (Accurus Vitrectomy System, Alcon Laboratories Inc, Fort Worth, TX)using standard 23-gauge vitrectomy techniques. Pars plana vitrectomy (PPV) was performed using a 23-G high-speed vitrector with a cut rate of 2500 per minute (Accurus Vitrectomy System, Alcon Inc., USA). The vitrectomy was performed from posterior vitreous-to-vitreous base. The posterior hyaloid was removed using active aspiration in cases without complete posterior vitreous detachment. Triamcinolone acetonide was used to ensure that the posterior hyaloid was lifted and removed in all of the cases. The aspiration power was 300 mmHg, 500 mmHg, and 150 mmHg, in core vitrectomy, active aspiration of posterior hyaloid and removal of the peripheral posterior vitreous, respectively. To remove the foreign body, one of the sclerotomy sites was enlarged like the T or L letters. With the 20-gauge forceps, the IOFB was removed without any difficulty. Endolaser treatment was applied with a curved 23-G laser probe (Iridex, Mountain View, CA) to the retinal entrance of the foreign body and other retinal breaks.

#### 23 G PPV and IOFB removal and vitreous hemorrhage

After the sclerotomies were placed vitreoretinal procedures included peeling of the posterior hyaloid membrane, endophotocoagulation, fluid-air exchange, and gas or silicone oil injection in appropriate cases were performed.

#### 23 G PPV and IOFB removal and retinal detachment

In patients with retinal detachment the vitrectomy begins with the removal of the vitreous humor, followed by displacement of the subretinal fluid by a heavy tamponade (perfluorocarbon) and scarring of the retina by laser coagulation. The vitreous is then replaced by a tamponade, which holds the retina against the underlying retinal pigment epithelium until a scar has formed around the retinal hole. As endotamponade, silicone oil was injected with a 23-gauge cannula system and 10 mL injector with an injection pressure in complex cases such as RRD with inferiorly located breaks, proliferative vitreoretinopathy, or severe TRD. Silicone oil was used as a tamponade in young adults; large holes; conditions preventing strict facedown position; or in patients unwilling to undertake the strict positioning otherwise gas tamponade was used.

At the end of surgery an absorbable 7-0-vicryl suture was used to close the expanded sclerotomy site and the conjunctiva. The remaining two 23-gauge micro cannulas were then removed and sclerotomy sites were also closed with 7-0-vicryl suture. Postoperative examinations were conducted at first day, first week, and at 1, 3, 6 months and last visit.

### Main outcome measures

Main outcomes were recorded including anatomic and visual outcomes, IOP, and both intraoperative and postoperative complications. Anatomical success was considered the total attachment of the retina at the end of the follow-up time. Totally or partially detached retina was considered as failure.

### Statistical analyses

Statistical analyses were performed using the Statistical Package for Social Sciences version 20.0 (SPSS Inc, Chicago, IL, USA). An assessment of normality was done initially. All numerical data are expressed either as the median (minimum-maximum) or as the mean ± _standard deviation. All categorical variables are expressed as the number and percentage (n, %). The Wilcoxon signed-rank test was used to compare the variables (Snellen visual acuity was converted into logarithm of the minimum angle of resolution (LogMAR) for statistical analysis) A *P*-value of, 0.05 was considered to be statistically significant.

## Results

### Demographical results

Thirty-six eyes of 36 patients were included in the case series. Their age was 34,2 ± 10,9 (15–60) years and they were followed for 9,4 ± 6,4 (2–27) months. The characteristics of patients are shown in Table 1. The characteristics of intraocular foreign bodies are shown in Table 2.

**Table 1:** Demographical data of patients

Demographics and clinical features (*n* = 36)		
Age	Years Mean ± SD, (Range)	34.2 ± 10.9
Gender	Female	9
	Male	27
Eye	Right	54.1 %
	Left	45.9 %
Interval^a^	Days Mean ± SD, (Range)	14.2 ± 19,4(1–120)
Follow-up	Months Mean ± SD, (Range)	9.4 ± 6.4(2–27)

^a^The time between the injury to surgery

**Table 2:** Characteristics of Intraocular Foreign Bodies

	Number (%)
IOFB entrance	
Corneal	26 (%72,2)
Scleral	10 (%28,8)
Corneascleral	0 (%0.0)
IOFB location	
Vitreus	20 (%55,6)
Retina	16 (%44,4)
IOFB type	
Metallic	28 (%78,8)
Stone	8 (%22,2)

### Previous surgeries and preoperative accompanying diseases

Seven cases had a primary wound repair and three patients had both primary wound repair and cataract extraction, which were performed by a previous medical center before being referred to our hospital. Six cases underwent a primary wound repair in our hospital before the 23-G TSV and IOFB removal. The remaining 20 cases did not require a primary wound repair. Accompanying diseases were traumatic cataract in 20 (55.6 %) cases, vitreous hemorrhage in 12 (16.6 %) cases, retinal detachment in 6 (16.6 %) cases, and endophthalmitis in one (2.7 %) case.

### Visual outcomes

Mean preoperative LogMAR BCVA was 1.44 ± 138 (range, 1.00 to 0.00) and mean postoperative LogMAR BCVA at final visit was 0.78 ± 0.98(range, 1.00 to 0.00) (*p* = 0,007). Ten patients *(%27,8)* final visual acuity were better than preoperative values (Table 3). Distribution of preoperative and final visual acuities were shown in Tables 3 and 4.

**Table 3:** Distrubition of preoperative and final visual acuities

	Preoperative, n (%)	Final visit, n (%)	*P* values
HM* or less	10 (%27,8)	4 (%11,1)	>0.05
CF**- < = 0.1	6 (%16,6)	10 (%27,8)	>0.05
>0.1- < = 0.3	6 (%16,6)	2 (%5,5)	>0.05
>0.3	16 (%44,4)	20 (%55,6)	>0.05

*HM* hand movement; *CF* finger count

**Table 4:** Baseline and final visit BCVA of patients based on IOFB characteristics

	Baseline BCVA^a^	Final Visit BCVA^a^	P values
IOFB entrance			
Corneal	1,22	0,80	0,029
Scleral	2,02	0,74	0,443
P^1^	0,568	0,183	
IOFB type			
Metallic	1,18	0,60	0,022
Stone	1,87	1,31	0,082
P^1^	0,127	0,100	
IOFB Location			
Retina	1,42	0,89	0,070
Vitreus	1,19	0,55	0,871
P^1^	0,077	0,470	

^a^LogMAR BCVA values. *P* values <0.05 was considered as significant

### Surgical procedures

Two patients underwent intraocular lens implantation in the sulcus combined with 23-G PPV and IOFB removal. A patient was left aphakic. Table 5 summarized the surgical procedures that applied to the patients. As endotamponade, silicone oil was injected in eight (22.6 %) patients and gas tamponade was injected in 14 (38.8 %) patients; the remaining 14 (38.8 %) patients were not injected any endotamponade. Seven of these patients underwent the silicone oil removal for a mean of 6.4 months (ranging from 4 to 8 months). At follow up, two (5.5 %) patients underwent re-vitrectomy due to retinal detachment and four (11.1 %) patients underwent phacoemulsification and IOL implantation. The mean extracted intraocular foreign body size was 5.63 mm (size range, 3 to 12 mm).

**Table 5:** Surgical procedures

	n (%)
Surgical	
23G PPV + IOFB removal	17 (47,2 %)
23G PPV + IOFB removal + PHACO + IOL imp	17 (47.2 %)
23G PPV + IOFB removal + IOL imp	2 (5,5 %)
Intraocular tamponades	
No	14 (38.8 %)
Silicone oil	8 (22,6 %)
Gas (C3F8/SF6)	14 (38,8 %)

### Intraocular pressure changes

The preoperative and postoperative IOPs were 12,1 ± 4,0 and 13,7 ± 4,0, respectively (*p* > 0,05).

### Anatomical results

At last follow-up visit, anatomic success was obtained in 97.2 % of eyes.

### Postoperative complications

Early postoperative period, a fibrin reaction was seen in eight (22.2 %) patients. Intraocular pressure elevation was detected in 12 (33.3 %) patients. All of the patients with intraocular pressure elevation had silicone oil as an intravitreal tamponade. Four (11.1 %) patients with intraocular pressure elevation were controlled with medical therapy and one patient underwent diod laser cylophotocoagulation. One of eight patients with silicone oil tamponade developed band keratopathy and phthisis bulbi.

## Discussion

Traumatic eye injuries associated with intraocular foreign bodies (IOFBs) may result in devastating tissue disruption and severe visual loss depending on a number of factors including the time between trauma and IOFB extraction, initial visual acuity, entrance wound location, nature of IOFB, location of IOFB, preoperative retinal detachment, presence of intraocular hemorrhage, presence of endophthalmitis, use of lensectomy, use of an encircling band, type of endotamponade, and primary surgical repair combined with IOFB removal and the occurrence of postoperative complications. The aim of treatment in IOFB is to restore the ocular integrity and obtain a good visual outcome.

The majority (59–88 %) of IOFBs were located in the posterior segment and the best management is pars plana vitrectomy [24, 25]. Recent advances in vitreoretinal surgery and microsurgical techniques, using intraocular tamponade, have improved the success rate of pars plana vitrectomy (PPV) in the management of ocular injuries with retained posterior segment IOFB [11, 13]. The current strategy to reduce the rate of secondary complications comprises the operative removal of the vitreous, including all proliferative mediators, and stabilization of the retina without remaining traction. However, even advanced vitreoretinal surgery cannot prevent recurrent proliferative vitreoretinopathy (PVR) with deleterious long-term outcome including phthisis bulbi.

The 23-G vitrectomy allows for increased comfort, faster healing time, reduced corneal astigmatism, shorter surgical time, rapid postoperative and visual recoveries, less inflammation and less disruption to the conjunctiva than with 20-gauge procedures [26, 27]. The smaller port size of the instruments increases the ability to remove vitreous with very little traction or to remove epiretinal membranes without risking incarceration of the retina in the port.

Currently, although many vitreoretinal surgeons have accepted small gauge vitrectomy for vitreoretinal diseases, usage of small gauge PPV is not widespread in the management of ocular injuries with retained posterior segment IOFB. There are a few published studies in the literature for small gauge vitrectomy and posterior segment IOFB removal. Kiss et al. reported that anatomic and visual outcomes of transconjunctival 25-gauge PPV for treatment of IOFB removal [28]. Kunikata et al. showed the usage of 25-Gauge micro incision vitrectomy surgery for removal of large IOFB in two cases [29]. However, there are few published studies on outcomes of 23-G PPV for posterior segment IOFBs [30].

In this present study, we evaluated the efficacy and safety of 23-gauge PPV for treatment of retained posterior segment IOFB. The mean BCVA improved significantly from 1.44 LogMAR to 0,78 LogMAR (*p* = 0,007) and an anatomic success was obtained in 97.2 % of eyes.

Traumatic cataract associated with IOFB is a common problem, which ranges from 44 to 66 % [31]. In our study, traumatic cataract was seen in 55.6 % of eyes. A combined vitreoretinal and cataract surgery was performed in these cases.

Retinal detachment associated with IOFB is the main reason for visual loss following intraocular foreign body injuries involving the posterior segment. Despite surgical advances in managing posterior segment, intraocular foreign body injuries, preoperative, and postoperative retinal detachments remain a frequent and devastating secondary complication. Rhegmatogenous retinal detachment after penetrating trauma is rapidly and severely complicated by proliferative vitreoretinopathy, and proliferative vitreoretinopathy was the reason for failure of retinal detachment surgery in the eyes that developed rhegmatogenous retinal detachment in other series [32, 33]. The rate of retinal detachment associated with IOFB ranged from 16 to 47 % [4, 21, 34]. In our study, preoperative retinal detachment was seen 16.6 % of eyes. Also, postoperative retinal detachments developed in two eyes (5.5 %). Scleral entrance and foreign bodies larger than 3 mm were associated with retinal detachment.

Visual loss may be associated with the timing of IOFB removal, scleral entrance of IOFB, preoperative visual acuity, and secondary complications such as endophthalmitis, rhegmatogenous retinal detachment (RD), intraocular pressure elavation, cataract, inflammation, or foreign body toxicity. In a study, early surgery, good preoperative visual acuity, and small IOFB were indicated as good prognostic factors [4, 35]. Our study showed that good preoperative visual acuity associated with good prognosis, retinal detachment and endophthalmitis were associated with worse prognosis, and IOFB size, location, and entrance were not associated with prognosis.

The size of the IOFB is one factor associated with postoperative anatomical and visual outcomes. The increasing size of IOFB was significantly associated with a poor visual outcomes [36]. The size of IOFB has been found to be a significant predictive factor of poor visual outcome in the previous studies of IOFB removal [37]. A large IOFB is more likely to inflict severe damage at the time of entry because of its higher kinetic energy, leading to a poor visual prognosis [37]. However, when considering similar sized IOFBs, no particular association between the visual outcome and the size of IOFB in eyes that developed endophthalmitis was found in our study. Despite these discrepancies in the influence in IOFB size on functional and anatomic outcomes, however, IOFB size does play a role in operative decision-making, and should be noted carefully. In our study, the mean size of IOFB was 5.63 mm. For removal of IOFB, a 23-G sclerotomy was expanded like the shape of the T or L letters.

Our study had some limitations. Our follow-up period was *9.4 ± 6.4 months*; longer follow up periods were necessary for more precise results on IOFB complications. Lack of the images for the preoperative and postoperative periods of the cases were our other main limitation in this paper. Nevertheless, our study is the first investigating the 23-G PPV with IOFBs.

## Conclusion

In conclusion, partial 23 gauge PPV and incision for IOFB removal appears to be an effective and safe procedure in the management of posterior segment IOFBs.
